# Factors affecting the physical activity of healthcare workers of Iran University of Medical Sciences: a qualitative study

**DOI:** 10.1186/s13690-022-00963-7

**Published:** 2022-09-22

**Authors:** Soodabeh Hoveidamanesh, Batool Tayefi, Zahra Rampisheh, Narjes Khalili, Mozhdeh Ramezani

**Affiliations:** 1 Burn Research Center, Iran University of Medical Sciences, Motahari Burns Hospital, Yasemi St., Vali-Asr Ave., Vanak Sq., Tehran, Iran; 2 Preventive Medicine and Public Health Research Center, Psychosocial Health Research Institute, Department of Community and Family Medicine, School of Medicine, Iran University of Medical Sciences, Shahid Hemmat Highway, Tehran, 14496-14535 Iran

**Keywords:** Physical activity, Healthcare workers, Iran

## Abstract

**Background:**

Low physical activity in adulthood is a major public health challenge. The majority of adults spend many hours each week at work, and workplace thus becomes a suitable location in which to promote health and implement physical activity programs. This qualitative study was conducted to identify the barriers and facilitators of worksite physical activity from the perspective of the employees of Iran University of Medical Sciences.

**Methods:**

In this qualitative thematic analysis, five focus group discussions were held with the participation of 68 staff members of Iran University of Medical Sciences who had been selected by purposive sampling with maximum diversity.

**Results:**

The analysis of the data led to the identification of three general themes, including challenges and barriers, strategies, and incentives (facilitators). The four main categories of challenges and barriers included policy-making and legislation, organizational factors, structural factors, and personal factors. Most barriers identified by the participants were placed in the personal factors and organizational factors categories. The strategies for increasing physical activity were identified in the following three categories: Policy-making and legislation, organizational factors, and environmental factors. The majority of the strategies proposed were placed in the organizational factors and policy-making and legislation categories.

**Conclusions:**

Increasing physical activity in the workplace as a strategy for the general promotion of physical activity in people requires interventions in different areas, especially with regard to organizational factors and policy-making and legislation.

## Background

Physical activity is defined as any movement of the body that requires energy expenditure [1]. Physical activity improves muscular and cardiorespiratory fitness and bone health and reduces the risk of hypertension, coronary heart disease, stroke, type 2 diabetes, various types of cancer, and depression [1–5]. A meta-analysis found that workplace physical activity interventions improve employees’ health and mitigate their work stress [6]. Physical inactivity, on the other hand, increases the risk of Non-Communicable Diseases (NCDs) by 20–30% as one of the main risk factors, and shortens individual lifespan by 3–5 years, increases the hidden costs of medical care, and decreases productivity [1].

In 2016, 23% of men and 32% of women above 18 years globally were reported to suffer from insufficient physical activity. The highest prevalence was 39% in the WHO region of the Americas and 35% in the Eastern Mediterranean region [7]. In Iran, a systematic review demonstrated the prevalence of physical inactivity as approximately 30% to almost 70% [8]. According to a Global Burden of Disease Study (GBD), from 1990 to 2017, Disability-Adjusted Life Years (DALY) attributable to low physical activity increased 1.5 and 2-fold globally and in Iran, respectively [9].

Despite its obvious health benefits, the amount of physical activity is still far from desirable, and low physical activity remains a public health challenge [2]. Through specific interventions, physical activity can become an integral part of workplace health promotion [5]. Based on a cohort study, sickness absenteeism decreases as leisure time physical activity increases but further increases as occupational physical activity increases [5].

In 2016, Iran’s STEPS survey revealed that work (53.7%) was the largest domain in which physical activity takes place in all age groups for both genders, followed by transport (33.6%) and recreation (12.8%), especially in 35–44 year old individuals and men [9]. The majority of adults spend many hours at work every week [10–12], and the workplace has thus been recognized as a suitable site in which to promote public health and raise awareness about the risk factors of NCD and to establish physical activity programs [6, 10–12]. Several studies have also suggested that workplace interventions, such as promoting stairway use, reducing sedentariness, and increase physical activity behaviors at work, and all aspects of daily life [11, 12].

Implementing workplace health programs can significantly improve the health status of the participating employees; however, the development and implementation of workplace health interventions requires a strong commitment on the part of the organizational leadership, an inclusive health culture, and the availability of the required resources and infrastructure [13]. Studies have emphasized the importance of organizational support strategies [12, 14, 15], and since organizations vary widely, flexibility is required in developing effective health plans for each organization in accordance with the needs of its employees [13].

This study was designed in 2019–2020 to develop strategies for increasing the physical activity of the employees of Iran University of Medical Sciences. This qualitative study was implemented as part of that study to identify worksite physical activity barriers and facilitators from the perspective of the employees.

## Methods

### Research methodology and paradigm

The present qualitative study is based on the naturalistic philosophy and interpretive paradigm and uses thematic analysis to analyze the data. This method seeks to understand and discover human experiences, since the structure of truth for each person is shaped by his own experiences [16].

The five-stage analysis method proposed by Graneheim and Lundman was used for the content analysis of the qualitative data. These stages included the transcription of each interview immediately afterwards, reviewing the entire text to obtain a general understanding of its content, determining the meaning units and initial codes, the classification of similar codes in more comprehensive categories, and determining the main theme of the categories [17].

### Research team members

The researchers were specialists in preventive medicine and community medicine, and two of the team members held the focus group discussions and coordinated the sessions.

### Sampling and sample size

Non-randomized purposive sampling was performed and the study continued until the saturation of data, when no further new codes could be extracted from the group discussions. For maximum variation sampling, the participants were selected from five main job categories, including university administrative/financial units, faculties, hospitals, health networks or healthcare centers of Iran University of Medical Sciences. A total of 51 participants (76.1%) were female.

To observe the maximum diversity, the participants were selected from different job categories.

### Participant’s characteristics

The present study was conducted with the participation of 68 people from different units of Iran University of Medical Sciences, including physicians, nurses, health service providers, headquarter staff, managers, and specialists. The participants were selected from those who were good at expressing their ideas, had intrinsic interest in expressing their experiences, and had fairly recent and recallable experience about the situation under study [18]. A face-to-face interview approach was adopted for the focus group discussions. No one withdrew from this study. The sampling process continued until the saturation of data, when no further codes could be extracted.

### Study setting

The focus group discussions were held in Malard Healthcare Network, the West Healthcare Center, Hazrat-e Rasool Hospital, Hasheminejad Hospital and the headquarters of Iran University of Medical Sciences. The interviews were conducted during work hours at participants’ workplace between 2019.08.31 and 2019.09.29.

### Data collection

After obtaining permission from the authorities, letters were sent to the directors of the centers and hospitals, asking them to send out the invitations to any eligible candidates, which also included explanations about the study objectives, questions to be posed in the meetings, and an application for participation in the study. Arrangements were then made and the interviewers presented to the specified locations. Data were collected by focus group discussions and semi-structured open-ended questions. Data were extracted from participants with maximum diversity in terms of demographic details and job categories, irrespective of their previous level of physical activity. The interview questions were based on a guide prepared for this purpose through a review of literature. Pilot interviews were conducted with three of the experts and the research team resolved any defects in the interview guide. Probing questions were also asked in the interviews if required, such as “Could you explain further?”, or “What do you mean by saying …?”

The interviews were conducted at participants’ workplace in a quiet and comfortable room. After introducing herself and her colleagues, explaining the study objectives, ensuring the participants of the confidentiality of the data, and obtaining their permission, the facilitator conducted the interviews. In each session, one person acted as the administrator and interviewer and another person took notes. Before asking the questions, the participants were asked to read the informed consent form and sign it if they wished to participate in the study. At the end, the participants were asked to discuss anything else they had to say. With the participants’ permission, the interviews were recorded. Each group discussion session lasted a minimum of 67 minutes and a maximum of 138 minutes.

### Data analysis

Data were analyzed concurrently with their collection using thematic analysis. This method is useful for identifying common and repeating themes, topics, and patterns [19]. The group discussions’ content was transcribed by a professional transcription company. The transcribed data were reviewed line by line for the analysis. Then, based on a proper schedule, the two researchers (S. H. and N. Kh.) carefully reviewed each interview text line by line at least twice and checked it against the recorded content to find meaning units from participants’ narratives. The meaning units were encoded and then classified, combined, and summarized based on their conceptual similarities. The extracted codes were discussed with the other researchers over two sessions. This process continued until the codes, subthemes, and main themes of the research were formed.

### Data rigor

Two of the researchers and another expert in qualitative research controlled the study process and confirmed the reliability of the data. The interview texts and the initial codes extracted were sent to the participants on which to comment; this step helped reinforce the transferability of the extracted data.

To ensure the trustworthiness and credibility of the data, a summary of the issues discussed for each question was given to the participants to approve or reject. To ensure confirmability, efforts were made to not allow the researchers’ assumptions to interfere with the process of data analysis. To ensure the transferability of the results and assess the data validity, effective communication based on trust (by explaining the study objectives clearly and ensuring the confidentiality and anonymity of the data) was established with the participants. Moreover, the participants were selected from various job categories to ensure maximum sampling variation.

## Results

Five focus group discussions were held with the participation of the personnel of Malard Healthcare Network, the West Healthcare Center, Hazrat-e Rasool Hospital, Hasheminejad Hospital and the staff of Iran University of Medical Sciences. A total of 68 people with a mean age of 40.7 ± 7.29 years participated in the focus group meetings. The mean duration of these meetings was 95 ± 35.76 minutes. Table 1 presents participants’ demographic details. The analysis of the data led to the identification of three general themes, including challenges and barriers, strategies, and incentives (facilitators), which are separately presented and explained in Tables 2, 3 and 4.

**Table 1:** Demographic characteristics of participants (*n* = 68)

Variable	Number (%)
**Gender**	
**Male**	**16 (23.9%)**
**Female**	**51 (76.1%)**
**Marital status**	
**Married**	**40 (61.5%)**
**Single**	**24 (36.9%)**
**Education**	
**Below high school diploma**	**1 (1.5%)**
**High school diploma & associate degree**	**7 (10.6%)**
**Bachelor’s degree**	**29 (43.9%)**
**Master’s degree**	**18 (27.3%)**
**PhD**	**11 (16.7%)**
**Work shifts**	
**Day**	**53 (84.1%)**
**Night**	**1 (1.6%)**
**Rotating**	**9 (14.3%)**
**Job category**	
**Health**	**28 (46.7%)**
**Medical**	**16 (26.7%)**
**Administrative/financial**	**12 (20%)**
**Faculty member**	**4 (6.7%)**

**Table 2:** The codes extracted for the challenges and barriers theme

Theme	Sub theme	Code
**Challenges and barriers**	**1.1. Policy-making and legislation**	**1.1.1 Employees’ physical activity not being a priority at the macro level**
		**1.1.2 Physical activity not being compulsory**
	**1.2. Organizational factors**	**1.2.1. Long work hours**
		**1.2.2. Large number of work shifts**
		**1.2.3. Playing multiple roles in the system**
		**1.2.4. Shortage of workforce**
		**1.2.5. Mechanization and computerization of tasks**
		**1.2.6. Allocating a bad time to exercise (after work hours)**
		**1.2.7. Type of work (working at a desk or computer)**
		**1.2.8. Stressful work environment**
		**1.2.9. Ruling organizational culture**
		**1.2.10. Personnel’s physical and mental health not being important for some managers**
		**1.2.11. Job burnout**
		**1.2.12. Poor notifications about the university’s sports and recreational programs**
		**1.2.13. The lack of motivation for physical activity**
	**1.3. Structural factors**	**1.3.1. The lack of physical space for exercise**
		**1.3.2. Distance (from work to home or home to the gym)**
		**1.3.3. Poor physical space unsuitable for performing physical activity (quality and size-wise)**
		**1.3.4. The lack of shower facilities at the university gym**
		**1.3.5. Old equipment and machines**
		**1.3.6. Lack of a coach**
		**1.3.7. The allocation of morning hours to women and evenings to men**
		**1.3.8. Marriage and raising children**
		**1.3.9. Personal and family culture**
		**1.3.10. Female gender**
		**1.3.11. The small space at home and the limitations of performing exercise in apartments**
	**1.4. Personal factors**	**1.4.1. The lack of motivation**
		**1.4.2. Exercise not being a priority in life**
		**1.4.3. Physical exhaustion**
		**1.4.4. Mental exhaustion**
		**1.4.5. Shortage of time (time constraints)**
		**1.4.6. Personal culture**
		**1.4.7. Having no exercise plans**
		**1.4.8. Financial and livelihood problems**
		**1.4.9. Laziness**
		**1.4.10. Family responsibilities**
		**1.4.11. The lack of awareness about the harms of a sedentary lifestyle**

**Table 3:** The codes extracted for the strategies theme

**Strategies**	**2.1. Policy-making and legislation**	**2.1.1. Creating and enforcing exercise breaks**
		**2.1.2. Allocation of budget to personnel’s physical activity at the macro level**
		**2.1.3. Raising awareness and engaging managers and officials in physical activity**
		**2.1.4. Making exercise mandatory for the personnel**
		**2.1.5. Incorporating physical activity into the civil service law**
		**2.1.6 Training and exercise programs in the workplace**
		**2.1.7. Promoting a culture of physical activity from childhood**
	**2.2. Organizational factors**	**2.2.1. Needs assessment and assigning an exercise liaison**
		**2.2.2. Hiring a coach**
		**2.2.3. Increasing the workforce size**
		**2.2.4. Reducing the work hours**
		**2.2.5. Holding physical activity training**
		**2.2.6. Reforming the organizational culture about the personnel’s physical activity**
		**2.2.7. Facilitating the health personnel’s transportation to and from the university gym**
		**2.2.8. Allowing different time slots for physical activity (before, during, and after work)**
		**2.2.9. The use of city bikes at the university, and morning jogs in groups**
		**2.2.10. Installing workplace exercise software on the personnel’s computers**
	**2.3. Environmental factors**	**2.3.1. Diversity in physical activity, and creating a suitable space for exercise**
		**2.3.2. Creating a suitable space for exercise, especially for women**
		**2.3.3. Standard, safe, easily-accessible exercise spaces**

**Table 4:** The codes extracted for the facilitators theme

**Incentives (facilitators)**	**3.1. Organizational factors**	**3.1.1. Giving gym passes or paying fitness subsidies and drafting contracts with gyms and pools**
		**3.1.2. The inclusion of physical activity in the personnel’s performance evaluation system**
		**3.1.3. Laying the groundwork**
		**3.3.4. Internalization and building a culture of exercise**
		**3.1.5. Inclusion of physical activity in the personnel’s health records**
		**3.1.6. Paying for exercise classes through the personnel’s wages**
		**3.1.7. Increasing the number of university gyms and sports facilities and improving the quality of the current gyms**
	**3.2. Motivational factors**	**3.2.1. Material and non-material incentives**
		**3.2.2. Physical activities in teams and groups**
		**3.2.3. The personnel going on walks in parks**
		**3.2.4. Holding ongoing and seasonal competitions**
		**3.2.5. Physical activity promoting campaigns**
		**3.2.6. Proper announcement of upcoming sports activities**
		**3.2.7. Creating desire and sensitivity**
		**3.2.8. Ensuring equity among the personnel from different departments**
		**3.2.9. Using NGOs for support**
		**3.2.10. Distributing educational pamphlets in the workplace to encourage the personnel to perform physical activity**
		**3.2.11. Diversity of sports fields**
		**3.2.12. Holding mountain climbing, hiking, and nature tours with the personnel’s family**
		**3.2.13. Hanging inspirational messages and quotes based on scientific evidence on the office walls to encourage physical activity**

### Challenges and barriers

This theme included four subthemes and 37 codes. The main four subthemes of challenges and barriers were policy-making and legislation, organizational factors, structural factors, and personal factors.

**Fig. 1:**
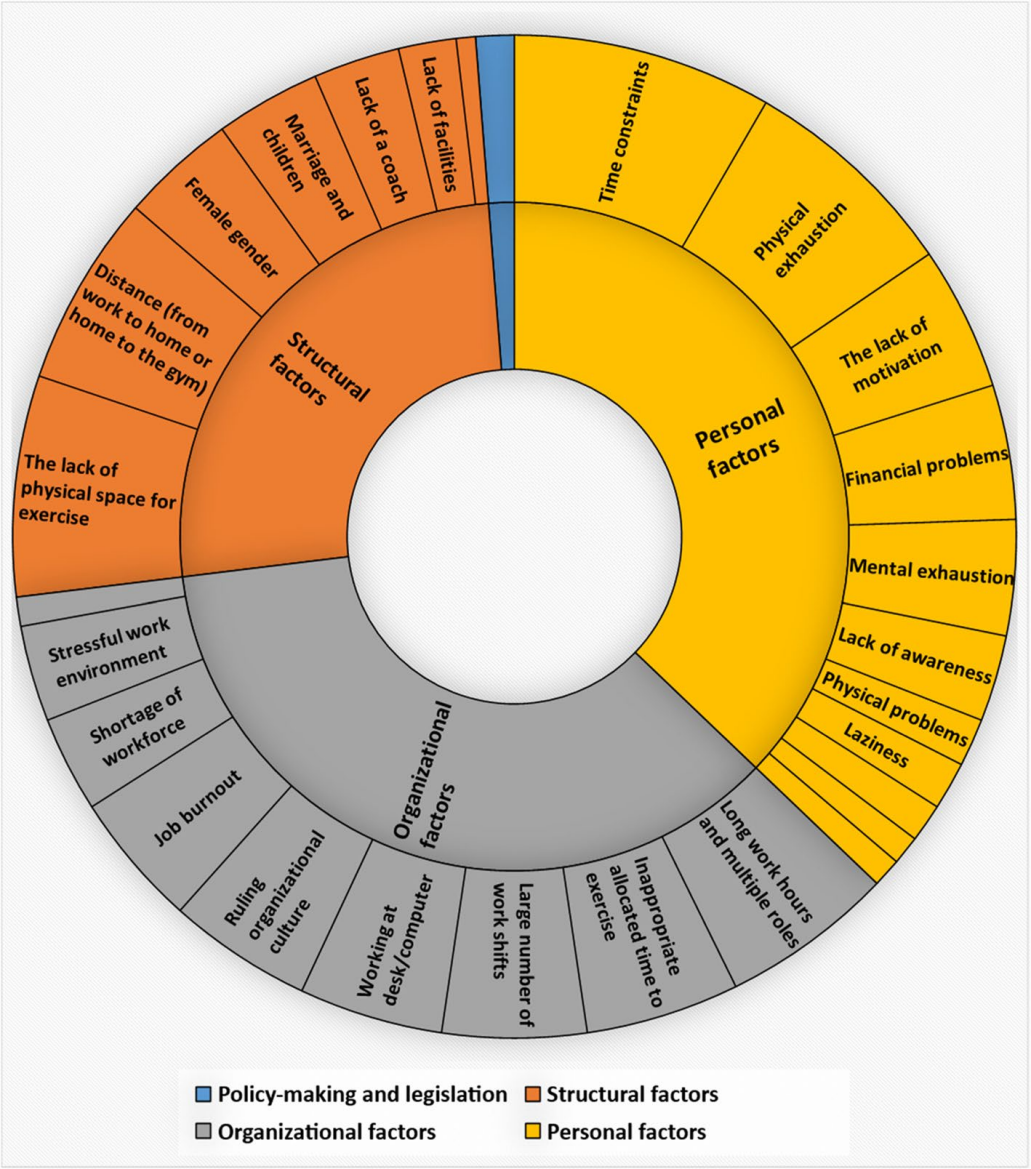
The most frequent extracted codes for the challenges and barriers theme

To identify the largest possible number of barriers to physical activity from participants’ view, the number of times each code in the barrier theme was repeated was counted. The majority of the codes pertained to the subtheme of personal factors, followed by organizational factors and structural factors. Only a few of the codes pertained to the subtheme of policy-making and legislation. Figure 1 presents the frequency of the codes repeated in this theme.

#### Policy-making and legislation

In the policy-making and legislation subtheme, some of the participants believed that an important barrier was that the personnel’s physical activity was not a priority for the managers and was not compulsory. “The main problem is that there’s no compulsory law on it. There must be some sort of obligation if you want to motivate people to do something; like, we have to work because it’s the law and we have to work to get paid” (P9).

The participants believed that the authorities prioritized only working, and the personnel’s physical activity and physical and mental health played no part in the job policies.

“The priority of the unit managers is for us to work –not engage in physical activity. If there was some sort of obligation, I mean, if a value system was established or if payments were made toward exercise in our salary” (P2).

#### Organizational factors

Regarding organizational factors, the most frequently-discussed items were organizational culture, desk work, heavy workloads, and large numbers of shifts. Moreover, long work hours, shortage of workforce, and being assigned multiple roles in the system were other issues emphasized by the participants.

“I personally believe that what has made me less physically active is that my job and work hours are excessive, and since I get very tired when I finally get to leave, I can’t handle much physical activity anymore” (P17).

#### Structural factors

The main barriers in the subtheme of structural factors that were emphasized by the participants included the lack of physical space for exercise or the poor conditions of the physical space for physical activity (both size-wise and quality-wise) and also the distance from home to the gym. The challenges faced by women when trying to perform physical activity, especially married women, constituted another reason for lack of physical activity in this category.

“If we want to use the gym facilities at the university after work, since the space is small, we have to wait for our turn on the machines” (P60).

“For example, if they said that we should gather round in the praying room every Monday to both have a chat and exercise in between, we would have agreed to join, but we were faced with restrictions instead. Well, our praying room is not just a praying room; it is also the lecture theater and the conference room. So, whenever we went there to exercise, others would want to come in after us and say their prayers” (P2).

“Well, most staff in health settings are female, married, and have kids –like myself. With kids, when I go home after work I have to pick them up from the kindergarten or school and then do the house chores. I have to handle all these, and then if I want to set aside some time for exercise, which I like very much, can I even? I love going to the gym, I might even plan for it, but I can’t really manage it” (P6).

#### Personal factors

In the subtheme of personal factors, the main barriers extracted in the present study included the shortage of time, physical and mental exhaustion, the lack of motivation, and financial problems.

“I think the main thing that makes us have no physical activity at all is the multiplicity of tasks assigned to us that fall beyond our line of work. We have forgotten ourselves altogether; we don’t show love toward ourselves at all and pay no attention to ourselves. Our seniors don’t care about us at all, like, do they ever ask if their personnel are physically and mentally healthy or not? We feel like we have been forgotten” (P19).

### Strategies

The strategies for promoting physical activity were classified into three subthemes and 19 codes, including policy-making and legislation, organizational factors, and environmental factors.

#### Policy-making and legislation

The findings related to this subtheme emphasized the development and implementation of exercise breaks, allocating funds to the personnel’s physical activity at the macro level, and raising awareness and engaging the managers and officials.

“There must be a law for this in the country, like the breastfeeding break that makes female employees actually get up and go to breastfeed their child. A period of, like, half an hour or twenty minutes should be legislated for this, and it should become the law, forcing employees to take it to exercise” (P14).

“There should be a budget specifically allocated to exercise, so we can dedicate a space for our colleagues. We must first have the space” (P15).

“If the organization and senior managers understood the necessity of it and if I wouldn’t be faced with the ramifications of not being at my desk for half an hour from, say, 10 to 10:30 in the morning, and if I didn’t have to be stressed over not being at my desk if my direct manager contacted me, then it would be easy” (P52).

#### Organizational factors

The strategies emphasized by the participants included needs assessment and assigning a sports liaison, recruiting a sports coach to teach the right exercises according to the employees’ physical conditions, increasing the workforce size to reduce work pressure and stress on the current employees, dedicating time to physical activities, and reforming the organizational culture.

##### Needs assessment and assigning a sports liaison

“For general and specialized exercises, I think there should first be a responsible expert liaison to assess the exercise needs of the personnel and see what their general or specialized needs are, and then based on those needs, the expert liaison should interview the people one by one; perhaps someone just can’t perform all the exercises and they are harmful for her” (P10).

##### Recruiting a coach

“A particular exercise may be suitable for a given person depending on his/her physical conditions, and the coach should advise the individuals on which physical activities to perform” (P59).

##### Increasing the workforce size

“As we said, we are short of manpower. We should reduce our work hours to have time for exercise. When I don’t have the power and strength, then I can’t prioritize exercise” (P11).

##### Reforming the organizational culture

“I think the organizational culture should be reformed, and we also must have a personnel health record. Everyone should think of exercise as a priority, and they should monitor their own progress and evaluate it, and think about exercise in both physical and mental terms, whether it is performed during work hours or outside the work hours, and the workload should also be somewhat adjusted to allow for it” (P9).

#### Environmental factors

The results in this section emphasized the need for diversity in physical activities, forming sports teams, building a standard and safe exercise arena for all the personnel, especially women, and ensuring easy access to the arena.

##### Diversity in physical activity and forming sports teams

“Team sports and group activities are good and make people energetic” (P9).

“Team sports are motivating. To create these teams, you should form groups and networks, make sports networks, and then choose one person as the leader to guide everyone” (P68).

##### Creating an appropriate place with easy access

“I think the university should dedicate a place to exercise –be it a gym, a pool, anything, so that everyone can exercise for free or for a small amount of money; if universities offer these facilities, everyone can have access to them and be physically active. Signing contracts with gyms close to each hospital or close to where the personnel live is also good” (P46).

### Motivators (facilitators)

The theme of motivators led to the extraction of organizational and motivational subthemes, with 7 and 13 codes, respectively.

#### Organizational factors

Most participants believed the main facilitators of physical activity to include the organization paying fitness subsidies, including the personnel’s physical activity in their performance evaluation, and building a ‘healthy and fit’ culture.

##### Paying fitness subsidies and drafting contracts with gyms and pools

“Paying fitness subsidies by drafting contracts with gyms and pools and giving the staff passes or subsidies and expanding the university gymnasium” (P60).

“If they draft a contract with some place and tell us that both men and women can go on certain days without imposing strict time slots, I think it will be welcomed by all the staff. But there is always the issue of costs, and we don’t expect it to be free of charge –just at a lower cost” (P46).

“The staff should be given passes to various gyms for free or half price, which should be financed by the ministry and not the network. The gyms should be located in different parts of Tehran, and using them should be obligatory, and the staff should be forced by their directors to use these facilities, and subsidies should be set aside to motivate the personnel” (P2).

##### Including the personnel’s physical activity in their performance evaluation

“An evaluation system should be defined for this, because there’s currently nothing in the civil service law at all; a payment should be considered for exercising on the payment scale; institutionalizing exercise, laying the groundwork for it and building a culture of physical activity are key. Our civil service law should be revised” (P11).

##### Laying the groundwork and building a culture of physical activity

“It has to do with the culture. Generally, we don’t feel the need neither at school nor in the family to perform physical activities. You grow up this way; you go to college and then to the workplace in this way, and then if one day they give me this opportunity [to exercise regularly], I don’t welcome it much. It has partly to do with the culture; its necessity has not been internalized. Now building a culture of physical activity is associated with knowledge. If I don’t know how to sit correctly, then I don’t sit correctly. It is the same with walking; I don’t walk correctly if I don’t know how to” (P55).

#### Motivational factors

The participants believed that building and improving motivation are key components of encouraging physical activity. Material and non-material incentives and holding competitions in areas such as mountain-climbing, hiking and nature tours with the family were some of the discussed measures.

##### Material and non-material incentives

“Giving points for exercise is one way. I mean to give points to someone who exercises rather than to deduct them from someone who doesn’t” (P60).

##### Holding competitions

“Another issue is that competitions should be ongoing. We only have few different occasions on which competitions are held, such as the anniversary of the Islamic Revolution, the ten-day celebration of Khomeini’s return to Iran, the government week, etc. But if these competitions were ongoing, if they were seasonal, in all fields possible, then I think it would be motivating. The personnel should all be allowed to take part in these competitions or hiking tours and they should be performed with respect for equity and fairness, so that everyone can take part” (P4).

##### Mountain-climbing, hiking, and nature tours with the family

“Creating a family atmosphere in which family members and friends can also attend these sports activities and turning them into group activities will certainly make them more effective” (P50).

## Discussion

The present qualitative study was conducted to identify the factors affecting physical activity in the personnel of Iran University of Medical Sciences to help design interventions to promote physical activity among university personnel. The content thematic analysis of the interviews led to the identification of three main themes, including challenges and barriers, strategies, and incentives (facilitators).

Challenges and barriers were categorized as policy-making and legislation, organizational factors, structural factors, and personal factors. In previous studies, barriers have been categorized into groups such as organizational views, operational outlook, and personal views [12] or into physical, psychological and environmental dimensions [6]. The various qualitative studies on this subject have found similar themes for the barriers to physical activity in the workplace, aside from these categories. The participants of the present study considered the shortage of time, physical exhaustion, and the lack of a suitable space for exercise as the biggest barrier to their physical activity, with the first two items being placed in the theme of personal factors. In other studies, the greatest barriers to exercise were noted as “having to invest time”, “fatigue” [20], “physical limitations due to pain and frailty”, “lack of motivation”, “lack of time”, and “job commitment” [6], “excessive exhaustion”, and “work commitment/long work hours” [21]. In some studies, the greatest barriers differed depending on the job category or type of work. For example, in one study, the managers recalled structural/organizational barriers, including regulations, costs, and the competitive aspect of work, while the employees tended to focus on personal limitations, such as the time and physical place for exercise [10]. As another example, employees in the transport industry, who are at a greater risk of inactivity compared to other jobs, regarded the changes in their work schedule, bad weather conditions, and the lack of planned holidays as the main barriers to their physical activity [22]. For midwives working in hospitals and health centers in Scotland, the barriers and facilitators of physical activity included fatigue, stress, family responsibilities, unpredictable rest hours and work shifts [23]. These items were more or less discussed similarly by the hospital staff surveyed in the present study. Some of these participants considered organizational culture a barrier to physical activity in the workplace and discussed particular cultural and other types of barriers in line with the study by Cooper et al. [24]. Similar to previous studies [6], psychosocial and environmental barriers appear to be a greater obstacle to physical activity than physical disorders.

Incentives (facilitators) were placed in organizational and motivational categories in our study. In previous studies, the most powerful incentives for physical activity have included family interactions, social support, the perceived health benefits of physical activity [6], subsidies given for exercise classes, and breaks given at specific times during work days [23]. The work environment and resources offered can be both a barrier and facilitator of physical activity [23]. In the present study, creating a space enabling exercise with family and friends was cited as a factor contributing to the motivation to exercise. Moreover, organizational incentives such as the payment of subsidies by the organization for exercise classes, including the personnel’s physical activity in the performance evaluation system, building a culture of exercise and creating motivation in the personnel to exercise were regarded as key incentives for performing physical activity. The results obtained by Brakenridge et al. show that workplace interventions supported by the organization are acceptable and can lead to long-term changes in awareness and culture [14]. Moreover, evidence-based interventions supported by key individuals as role models can spread to other workplaces [14]. Similar to in previous studies [25], some of the participants in the present study stated that urban design should be such that access to suitable spaces for playing sports is facilitated.

In the present study, the strategies for increasing physical activity were placed in three subthemes, including policy-making and legislation, organizational factors, and environmental factors. In Planchard’s study, interventions were classified as preparation, empowerment, reinforcement, environmental factors and policies [6].

Workplace wellness programs promoting physical activity can help businesses create effective policies and programs to meet their employees’ and businesses’ priorities [11]. In line with previous studies [23], the present study showed that interventions should focus on improving interpersonal relationships, reducing workplace stresses, and increasing social support. Just like previous studies [26], the present findings emphasized the need to pay further attention to women, especially working mothers, because the needs of this group should be further addressed through health counseling [26].

Although most barriers discussed by the participants were placed in the personal factors category, the majority of the strategies were related to organizational factors and policy-making and legislation categories. For instance, the lack of awareness about the risks of inactivity, which falls in the personal factors category of the theme of barriers, can be resolved by holding physical activity training courses, which falls in the organizational factors category of the theme of strategies. Various studies, including the present one, have emphasized the importance of organizational support strategies [12, 14, 15], even though multiple studies have also discussed organizations’ shortfalls in addressing these issues. For instance, in a study by Bailey et al. on the policies supporting physical activity in the workplace, a small number of the examined organizations had a written policy for increasing physical activity and allocating time to exercise during work hours, and the lack of such policy was described by the participants as a barrier to greater physical activity in the personnel [11]. In another study, Chau et al. investigated the views of the personnel of 12 different organizations and concluded that physical activity has not been a priority in these organizations’ occupational health programs [10]. Physical activity must be supported from all levels of an organization in order to make it an integral part of daily work [10].

The limitations of this study include restricting the data collection to focus group discussions, even though individual interviews could have provided more information about the personal barriers to physical activity. Nevertheless, the participants of this study worked in different centers and had limited time at their hands, which made it difficult to coordinate and conduct individual interviews. Also, we did not have sufficient financial resources and manpower for holding elaborate individual interviews. To understand the perspectives of the employees, the focus group method was chosen for data collection in this study because it encourages everyone to participate in a discussion guided by a facilitator (i.e., the researcher), and we wanted to know the most important barriers and facilitators. To sum up, we might have missed some information about the personal barriers. Moreover, moderator bias and social desirability bias are other potential limitations of this study.

A strength of this study that somewhat makes up for the discussed limitation is holding several focus group meetings in different university departments to ensure the maximum diversity of views and examine the subject from the perspective of administrative, health, medical, and headquarter staff alike.

## Conclusion

The results of our study show that barriers and facilitators fall into different categories. Although most barriers discussed by the participants were placed in the personal factors category, followed by the organizational and structural categories, the majority of the strategies discussed were related to organizational factors and policy-making and legislation categories. Therefore, strategies and interventions to promote physical activity in the workplace must cover different areas, especially areas related to organizational factors and policy-making and legislation. The researchers therefore recommend developing a written program to address the lack of physical activity among university personnel with an emphasis on strategies in different areas, especially in relation to organizational factors, while also taking into account the facilitators.

Some strategies for removing the barriers, such as holding training programs and installing workplace exercise software on the personnel’s computers, can be implemented at a negligible cost. These programs can raise managers’ and authorities’ awareness and engage them in promoting workplace physical activity programs.

## Data Availability

The datasets generated and/or analyzed during the current study are not publicly available due to confidentiality but are available from the corresponding author on reasonable request.
